# SORBS1 Knockdown Resists S/G2 Arrest and Apoptosis Caused by Polyphyllin H-Induced DNA Damage in Pancreatic Cancer

**DOI:** 10.32604/or.2025.064454

**Published:** 2025-08-28

**Authors:** Xinxin Hu, Yuye Xue, Fei Fang, Jie Li, Xiaofeng Yuan, Guang Cheng, Hailong Yuan, Yongqiang Zhang, Yuefei Zhou, Shuangwu Yang, Pengcheng Qiu, Yunyang Lu, Haifeng Tang

**Affiliations:** 1 School of Pharmacy, Shaanxi University of Chinese Medicine, Xianyang, 712046, China; 2 Department of Chinese Materia Medica and Natural Medicines, School of Pharmacy, The Fourth Military Medical University, Xi’an, 710032, China; 3 Department of Pharmacy, Air Force Medical Center, PLA, The Fourth Military Medical University, Beijing, 100142, China; 4 Department of Central Laboratory, The First Affiliated Hospital of Northwestern University, The First Hospital of Xi’an, Xi’an, 710069, China; 5 Department of Neurosurgery, Xijing Institute of Clinical Neuroscience, The Fourth Military Medical University, Xi’an, 710032, China

**Keywords:** Natural product, saponin, tumor, drug resistance

## Abstract

**Objectives:**

The Sorbin and SH3 domain containing 1 (SORBS1), a protein linked to insulin signaling CBL interaction, was investigated for its role in pancreatic cancer apoptosis. This study explored polyphyllin H (PPH)’s ability to restore SORBS1-knockdown-mediated repair functions.

**Methods:**

PANC-1 cells were divided into Blank, overexpression (OE), and knockdown groups. CCK-8 assays assessed proliferation and drug toxicity. Western blot and flow cytometry analyzed SORBS1 levels and PPH effects. Comet assays quantified DNA damage. Subcutaneous xenograft tumors in nude mice (Blank vs. knockdown) were treated with PPH to evaluate *in vivo* efficacy. SORBS1-H2AX gene correlation was analyzed Spearman rank clustering (*p* < 0.05).

**Results:**

PPH suppressed pancreatic cancer growth *in vitro*/*vivo*, but its efficacy was attenuated by SORBS1 downregulation. Clinically, low SORBS1 correlated with poor prognosis. SORBS1 knockdown promoted tumor proliferation and reduced PPH-induced apoptosis. While PPH decreased tumor volume in both Blank and knockdown groups compared to controls, SORBS1 knockdown diminished PPH’s inhibitory effects. Mechanistically, SORBS1 depletion mitigated PPH-triggered DNA damage, circumventing G2/M arrest by modulating WEE1, Cyclin A2, CDK1, and Cyclin B1, thereby impairing apoptosis.

**Conclusion:**

SORBS1 knockdown counteracts PPH-mediated S/G2 arrest and apoptosis by alleviating DNA damage in pancreatic cancer. These findings highlight SORBS1 as a critical modulator of PPH’s therapeutic potential, linking its expression to chemoresistance mechanisms.

## 1 Introduction

Although oncology has witnessed notable progress in recent years, pancreatic ductal adenocarcinoma, which makes up more than 90% of pancreatic cancers, is an extremely aggressive tumor with a bleak five-year survival rate of under 8% [1]. This highlights the urgent need for the identification of novel therapeutic agents and targets. Natural products have demonstrated potent antitumorigenic activities, rendering them a crucial source for discovering novel drugs and targets. Among them, saponins are recognized for their broad-spectrum antitumorigenic effects. Previous studies have shown that saponins isolated from various plants and animals [2,3], including Paris *polyphylla*, possess antitumorigenic properties [4,5]. Polyphyllin saponins primarily comprise steroidal compounds such as polyphyllin I, II, VI, VII and H. Their chemical structure is characterized by a spironane or isospironane parent nucleus, with sugar chains (e.g., glucose and rhamnose) attached glycosidic bonds. Mechanistically, saponins alter the expression of numerous genes in cancers. While some of these changes enhance anti-tumor efficacy, others may confer resistance. However, the majority of studies have focused on enhancing antitumor efficacy, with fewer addressing resistance mechanisms [6–8]. Investigating resistance-conferring genes could provide valuable insights for more precise therapeutic interventions.

Sorbin and SH3 domain-containing 1 (SORBS1) is essential for the tyrosine phosphorylation of Casitas B-lineage lymphoma (CBL) by connecting it to the insulin receptor. As is well documented, it is essential for insulin-stimulated glucose transport and is implicated in the formation of actin stress fibers and focal adhesions. SORBS1 has been identified in several cancers. In breast cancer, reduced levels of SORBS1 are linked to poor outcomes, with the PI3K/AKT pathway being notably enriched [9]. In bladder cancer, SORBS1 expression is significantly downregulated and may function as a tumor suppressor [10]. In colorectal cancer, it inhibits cancer growth and migration [11]. However, the role of SORBS1 in pancreatic cancer, particularly its interaction with saponin efficacy, remains to be elucidated. This study aimed to use polyphyllin H (PPH) as a probe to detect the function of repair of SORBS1-knockdown in pancreatic cancer apoptosis.

## 2 Materials and Methods

### 2.1 Materials

Polyphyllin H (PPH) was purchased from Must Biotechnology (Chengdu, China, Cat. No.: A1643, purity: 99.27%). The CCK-8 kit was provided by Elabscience (Wuhan, China, Cat. No.: E-CK-A362). The Comet Assay kit was procured from KeyGene bioTECH (Nanjing, China, Cat. No.: KGA1302-50), and lentivirus particles were sourced from Genechem (Shanghai, China). Dulbecco’s Modified Eagle Medium (DMEM) and fetal bovine serum (FBS) were obtained from Procell (Shanghai, China). The human pancreatic cancer cell lines PANC-1 and SW1990 [12] were acquired from the Cell Bank of the Chinese Academy of Sciences (Shanghai, China) and authenticated by Procell (Wuhan, China). Two cell lines have been STR-identified and tested for mycoplasma contamination. The primary antibodies used in this study included anti-SORBS1 (product code: 13854-1-AP, 1:500), anti-β-actin (product code: 66009-1-Ig, 1:10,000), anti-Cyclin A2 (product code: 66391-1-Ig, 1: 500), anti-CDK2 (product code: 60312-1-Ig, 1:500), anti-CDK1 (product code: 67575-1-Ig, 1:500), anti-Cyclin B1 (product code: 67686-1-Ig, 1:500), anti-γH2AX [Phospho-Histone H2A.X (Ser139), product code: 29380-1-AP, 1:400], and anti-WEE1 (product code: 29474-1-AP, 1:1000), all of which were procured from Proteintech (Wuhan, China).

### 2.2 PPH Toxicity Assay

Two cell lines, namely PANC-1 and SW1990, in log-phase growth were cultured in 96-well plates (1 × 10^4^ cells/well density) for subsequent experimental interventions. After 24 h, the cells were treated with varying concentrations (32, 16, 8, 4, 2, 1, and 0.5 µM) of PPH for 24 h. For each experimental condition, six replicate wells were established. A CCK-8 assay was used to evaluate cell proliferation and cytotoxicity after treatment. Specifically, a volume of 10 µL of CCK-8 reagent was placed in each well, and the mixture was incubated at 37°C with 5% CO_2_ for an hour. OD_450_ measurements were performed on a microplate reader (AMR 100, Hangzhou Allsheng Instrument Co., Ltd., Hangzhou, China) according to manufacturer protocols. Use the standard equation, which fits the data to a sigmoidal curve. Click “OK” to perform the nonlinear regression. The Graphpad Prism 9.0 will automatically calculate the IC_50_ value, 95% confidence intervals, and curve-fitting parameters.

### 2.3 Cell Culture and Lentiviral Infection

Basal SORBS1 expression was initially queried through the Human Protein Atlas (www.proteinatlas.org), and PANC-1 exhibited higher SORBS1 mRNA levels than SW1990. Two cell lines were randomly allocated to three groups: SORBS1-blank (infected with a non-targeting viral vector), SORBS1 overexpression (SORBS1-OE; infected with a viral vector designed to upregulate SORBS1), and SORBS1 knockdown (infected with a viral vector expressing SORBS1-specific shRNA). The efficiency of lentiviral infection was assessed using fluorescence microscopy (BX63, Olympus, Tokyo, Japan) after 3 days of culture. The target seq of SORBS1-specific shRNA was GCAGCAATGGGCAAGACAAAG, the order of elements of the viral vector expressing SORBS1-specific shRNA was hU6-MCS-Ubiquitin-mCherry-IRES-Neomycin, and the order of elements of the viral vector designed to upregulate SORBS1 was pRRLSIN-cPPT-SFFV-MCS-EF1N-dsRED2-SV40-neomycin.

### 2.4 Cell Proliferation Assay

Two cell lines, each assigned to three groups as described in the section “Cell Culture and Lentiviral Infection”, were distributed into four separate 96-well plates, each well holding 2 × 10^3^ cells per well during their logarithmic growth phase. Each individual plate was inoculated with cells at 24 h, 48 h, 72 h, and 96 h marks, respectively. For each experimental group, three replicate wells were set up. After the designated incubation times, 10 µL of CCK-8 solution was added to each well, and the resulting mixture was incubated for 1 h at 37°C in 5% CO_2_. Lastly, OD_450_ measurements were performed on a microplate reader according to manufacturer protocols.

### 2.5 Construction of the Pancreatic Cancer Subcutaneous Tumor Animal Model

The animal experiments in this study were approved by the Laboratory Animal Science and Technology Ethics Committee of Fourth Military Medical University (approval number: 240552). PANC-1 cells were maintained in DMEM containing 10% FBS at 37°C under 5% CO_2_ conditions. Cells were harvested upon reaching 70–80% confluence, washed with phosphate-buffered saline (PBS, pH 7.4), and resuspended to prepare a 1 × 10^8^ cells/mL single-cell suspension. The animal was a homologous inbred strain of BALB/c background carrying the naked gene (nu), a recessive mutant gene on mouse chromosome 8, mainly a spontaneous mutation in the Foxn1 (forkhead box N1) gene (formerly Hfh11 gene), purchased from Spfbiotech (Beijing, China). Then, nude mice received a subcutaneous injection of 100 μL of the cell suspension into their flank and were equally divided into four groups according to body weight: SORBS1-blank, SORBS1-blank + PPH, SORBS1-knockdown, and SORBS1-knockdown + PPH groups, with five nude mice in each group, totaling 20 6-week-old male nude mice. In order to follow the 3R principle, the comparison in this experiment only involved the comparison of drug effects between SORBS1-blank and SORBS1-knockdown groups, so there was no corresponding control group for each experimental group. Our previous pilot study showed that all five mice could survive at this dose. Next, on alternate days, 2.5 mg/kg PPH was injected intraperitoneally into nude mice. 717.55 µM PPH solution was prepared by dissolving PPH in a sterile PBS solution, with the safe dosage determined based on the study by Li et al. [13]. The study did not involve the inclusion or exclusion of animals, so no criteria were established. Xinxin Hu was aware of the group status at different stages of the experiment.

Situation. After 11 days, following euthanasia with CO_2_, the subcutaneous tumors of the nude mice were excised and measured. Thereafter, to prepare 4 µm-thick sections, tumor samples were fixed in 4% paraformaldehyde and then embedded in paraffin. Lastly, the expression level of γH2AX was detected using immunohistochemical staining.

### 2.6 Cell Apoptosis Analysis

PANC-1 cells, divided into three groups [SORBS1-blank, SORBS1 overexpression (SORBS1 OE), and SORBS1 knockdown], were cultured during their logarithmic growth phase and incubated for 24 h. Following incubation, the cells entered the exponential growth phase, they were treated with 2 μM PPH. Cells were sequentially collected after an additional 24 h, digested and washed with PBS. Post-treatment apoptosis was evaluated as follows: cells were dual-stained with Annexin V-FITC (PI, Cat No.: HY-D0815, MCE, Monmouth Junction, NJ, USA). Samples were analyzed immediately on a BD FACSCalibur system (Franklin Lakes, NJ, USA) equipped with a 15 mW argon laser. Apoptotic fractions were determined by quadrant analysis, excluding debris (FSC/SSC gating).

### 2.7 Cell Cycle Analysis

PANC-1 cells, divided into three groups [SORBS1-blank, SORBS1 overexpression (SORBS1 OE), and SORBS1 knockdown], were cultured during their logarithmic growth phase and planted for 24 h. Prior to flow cytometric analysis, cells were reared in serum-free DMEM at 37°C with 5% CO_2_ overnight. Subsequently, they were cultured for no more than 2 h in DMEM replenished with 10% FBS under identical circumstances. Cell cycle analysis used an Annexin V staining kit (Beyotime, Cat No.: C1062S, Shanghai, China), including Annexin V-FITC, PI staining solution, and 1 × binding buffer. The FCS file [FCS files were generated in real time during EXP032 software (Mindray, Shenzhen, China), collecting cells’ information] was exported into MultiCycle software (version 5, Phoenix Flow Systems, Eugene, OR, USA) for cell cycle analysis.

### 2.8 Western Blot Analysis

To explore the molecular basis of this resistance and assess the extent of SORBS1 downregulation, the western blot analysis has been used. Standard protocols were followed to conduct Western blot analysis. Briefly, cells were exposed to varying concentrations of PPH for 24 h and then lysed on ice for 30 min using RIPA lysis buffer (KeyGene bioTECH, Nanjing, China, Cat. No.: GKP2100). Total protein concentration was determined by BCA quantification. The samples were separated by sodium dodecyl sulfate-polyacrylamide gel electrophoresis (SDS-PAGE) and then transferred onto polyvinylidene difluoride (PVDF) membranes. Afterward, the membranes were blocked and then kept overnight at 4°C with primary antibodies against SORBS1, β-actin, Cyclin A2, CDK2, CDK1, Cyclin B1, and WEE1. The membranes were washed five times with PBST, each wash lasting 5 min, and then incubated with the corresponding secondary antibody for an hour. The Bio-RAD System (Hercules, CA, USA) was used to visualize protein bands after washing. Results were analyzed with Image Lab Software (v6.2, Hercules, CA, USA) for PC Version 6.1 (*n* = 3).

### 2.9 Comet Assay

PANC-1 cells, divided into two groups (SORBS1-blank and SORBS1-knockdown), were seeded into four dishes during their logarithmic growth phase and incubated for 24 h. Upon reaching the logarithmic growth phase, both the SORBS1-blank and SORBS1-knockdown groups were treated with 2 μM PPH. The cells were centrifuged and resuspended in PBS at a density of 1 × 10^6^ cells/mL after 24 h. For the Comet assay, the frosted side of the slides was used. Prepare 100 μL of 1% normal melting point agarose (NMA) solution and slowly add it to the slide, quickly cover it with a cover slip pre-cooled on ice, quickly place it in a 4°C refrigerator to solidify for 10 min, then carefully remove the cover slip, and then mix 10 μL of cell suspension (about 10^4^ cells) with 75 μL of 0.7% low-melting point agarose (LMA) solution. Quickly drop on the first layer of agarose, immediately cover the pre-cooled cover slip and set rapidly at 4°C for 10 min. Then carefully remove the cover slip, add 75 μL of dissolved 0.7% LMA solution, cover the pre-cooled cover slip and set at 4°C for 10 min. After lysis with the addition of precooled lysis buffer (Comet Assay kit, KeyGene bioTECH, Nanjing, China, Cat. No.: KGA1302-50) for an hour, the cells were rinsed with PBS, a newly made alkaline electrophoresis buffer was introduced, and the cells were electrophoresed at a 25 V level for 30 min. After removal, the slides were rinsed and neutralized with PBS three times, each for 10 min. The coverslips were removed and stained with 20 μL PI staining solution. Under the fluorescence microscope (BX63, Olympus, Tokyo, Japan) with excitation light of 555 nm, the nuclear DNA and migrating DNA (comet tail) could be clearly displayed. Randomly, one hundred cells were selected from each sample, and nuclear DNA diameter and DNA migration length were measured by comet analysis software Project (CASP 1. 2. 3 beta1, Wroclaw Medical University, Wrocław, Poland).

### 2.10 Gene-Gene Correlation Analysis

RNA-seq raw count data and corresponding clinical data related to pancreatic cancer were obtained from the Cancer Genome Atlas (TCGA) database (https://portal.gdc.cancer.gov). The expression values of transcripts were converted into transcripts per million (TPM) format and normalized using a log_2_ (TPM + 1) transformation. After filtering samples with incomplete RNA-seq or clinical records, 179 cases were ultimately selected for selected for further analysis. Pairwise correlations between genes were visualized using the ‘ggstatsplot’ package (v0.12.1) in R (v4.2.1), incorporating statistical significance annotations. Heatmaps for multi-gene correlation patterns were generated with the ‘pheatmap’ package (v1.0.12) (R v4.2.1), the hierarchical clustering based on Spearman’s rank correlation coefficients has been utilized. To examine correlations among quantitative variables lacking a normal distribution, Spearman’s correlation analysis was performed. Statistical significance was assigned to a two-tailed *p*-value under 0.05.

### 2.11 Statistical Analysis

All experimental procedures were conducted with biological replicates, with the majority of assays incorporating a minimum of three technical replicates (*n* = 3), as detailed in the corresponding figure legends. To ensure objectivity, investigators remained blinded to group allocations during data acquisition phases for western blot analysis, immunohistochemistry, PathScan multiplex detection, and flow cytometry experiments, with blinding maintained until completion of data collection. Levene’s test was used to verify the uniformity of variance before conducting parametric analysis. Statistical comparisons were made using either one-way or two-way ANOVA, with post hoc Tukey’s or Bonferroni’s tests applied as needed. Experimental data were processed and visualized using GraphPad Prism 9.0 (GraphPad, San Diego, CA, USA), with continuous variables presented as mean ± standard deviation. A threshold of *p* < 0.05 was established a priori for determining statistical significance.

## 3 Results

### 3.1 Low-Concentration PPH Inhibited Cell Proliferation and Down-Regulated SORBS1 Expression in Pancreatic Cancer

**Figure 1:**
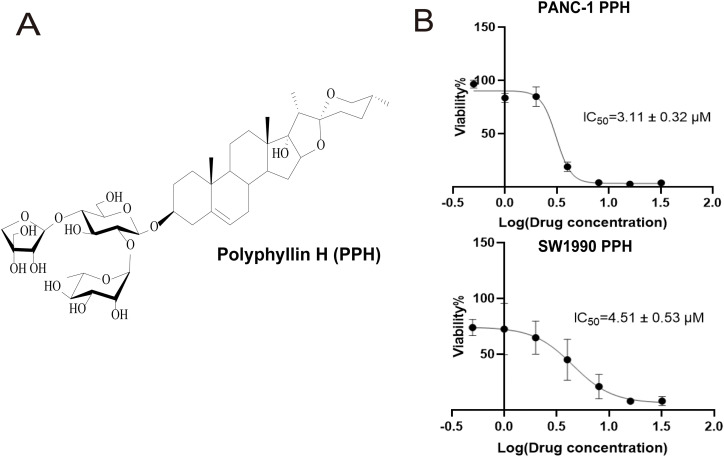
The molecular formula of Polyphyllin H (PPH) and its low concentration inhibition in pancreatic cancer cells (**A**) The molecular formula of PPH; (**B**) IC_50_ of Polyphyllin H (PPH) in PANC-1 and SW1990 cells (*n* = 3, 24 h, 95% likelihood)

**Figure 2:**
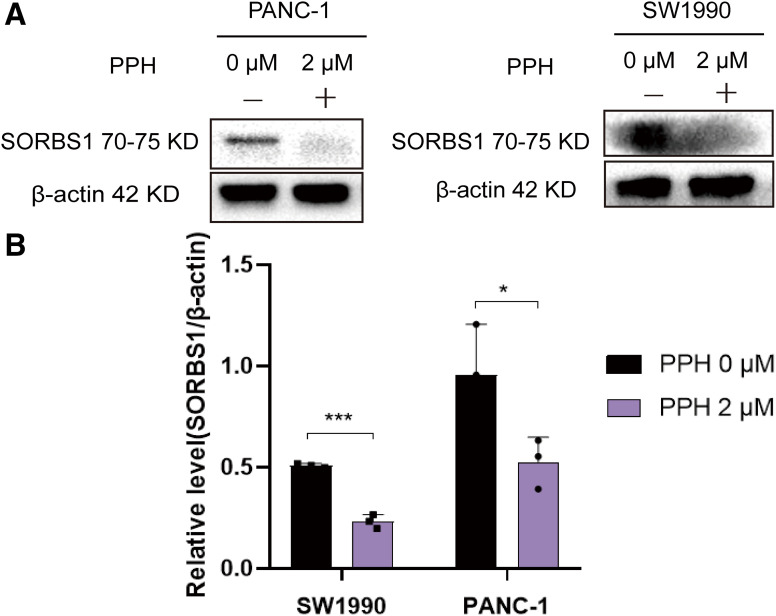
Western blot indicated PPH suppressed SORBS1 expression. (A) Western blot indicated PPH suppressed SORBS1 expression; (B) PPH downregulated SORBS1 protein expression in two cell lines by Western blot at 24 h (*n* = 3; *p < 0.05, ***p < 0.001 compared with 0 μM PPH group; “+” represents positive, and “−” represents negative compared with SORBS1-blank group)

PPH, isolated from *P. polyphylla* var. *stenophylla* (Fig. 1A), the inhibition of pancreatic cell growth in a dose-dependent way. To assess the connection between PPH and pancreatic cancer, two human pancreatic cancer cell lines were exposed to PPH concentrations of 0, 0.5, 1, 2, 4, 8, 16, and 32 µM. A CCK-8 assay was used to evaluate cell viability following 24 h of treatment, revealing IC_50_ (half maximal inhibitory concentration) values of 3.11 ± 0.32 μM for PANC-1 cells and 4.51 ± 0.53 μM for SW1990 cells (Fig. 1B). SORBS1 was significantly downregulated, consistent with the results of Western blot analysis (Fig. 2).

### 3.2 Low SORBS1 Expression Was Correlated with a Poor Prognosis and Increased Cell Proliferation in Pancreatic Cancer

**Figure 3:**
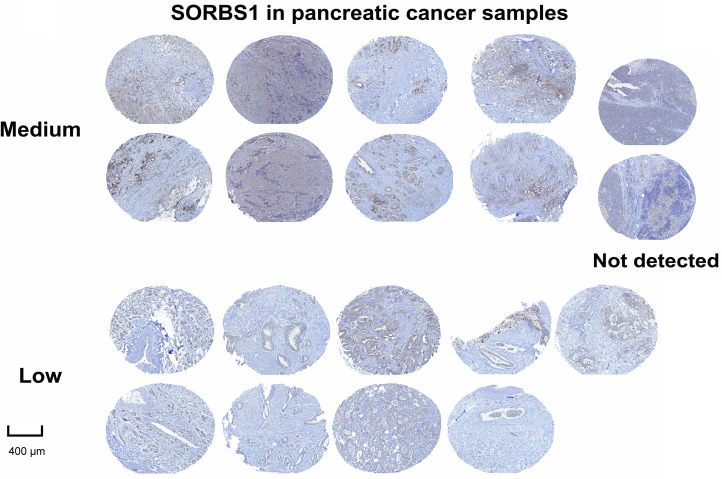
Immunohistochemical staining of SORBS1 protein expression showed the protein was at medium, low, or even undetectable levels in pancreatic cancer tissues collected by the Human Protein Atlas

**Figure 4:**
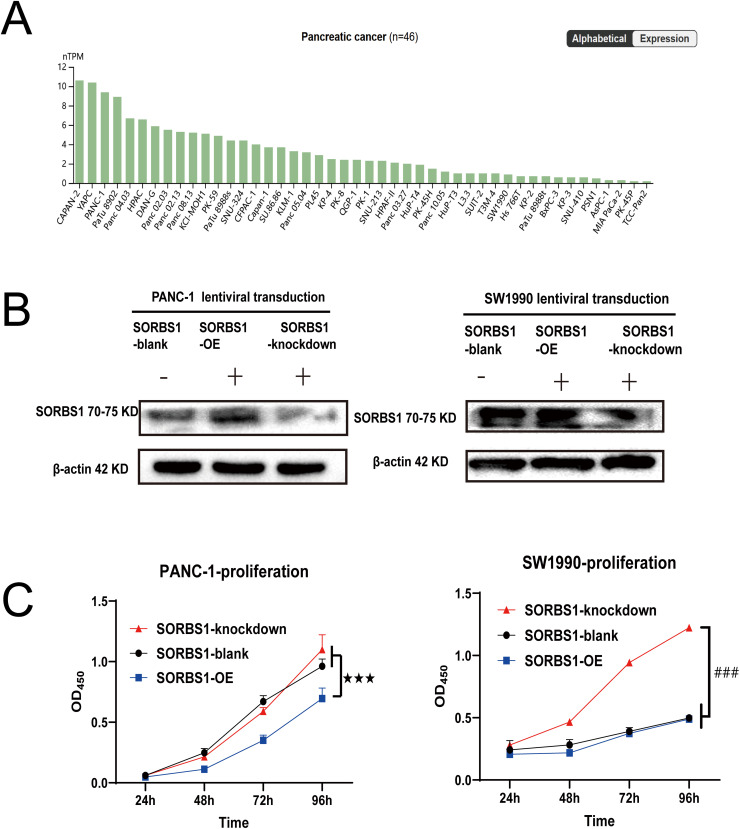
SORBS1 knockdown promoted pancreatic cell proliferation. (A) Basal SORBS1 expression was initially queried through the HPA portal (www.proteinatlas.org), PANC-1 exhibited higher SORBS1 mRNA levels than SW1990; (B) Western blot verified the efficiency of SORBS1 lentiviral infection in two cell lines (“+” represents positive, and “−” represents negative compared with SORBS1-blank group); (C) CCK-8 assay indicated differences in proliferation of different levels of SORBS1 in the two cell lines, respectively. (*n* = 3; 24 h; ^★★★^*p* < 0.001 compared with SORBS1-overexpression group; ^###^*p* < 0.001 compared with SORBS1-knockdown group)

SORBS1 was moderately or lowly expressed or even undetectable in pancreatic cancer tissues (Fig. 3). However, the involvement and mechanisms of SORBS1 in pancreatic cancer are not well-defined. To evaluate the relationship between SORBS1 and pancreatic cancer, pancreatic cancer cell lines were selected and infected with lentiviruses to alter SORBS1 expression levels. The selection of PANC-1 and SW1990 cell lines was based on their baseline SORBS1 expression levels in pancreatic cancer cell lines, as reported by the Human Protein Atlas (Fig. 4A). CCK-8 assays demonstrated that SORBS1 knockdown promoted the proliferative ability of two different cell lines; conversely, SORBS1 overexpression suppressed their proliferative capacity of two different cell lines (Fig. 4B). The efficiency of SORBS1 knockdown and overexpression was further validated by Western blot analysis (Fig. 4C).

### 3.3 SORBS1 Knockdown Attenuated the Inhibitory Effects of PPH on Pancreatic Cancer In Vitro and Vivo

**Figure 5:**
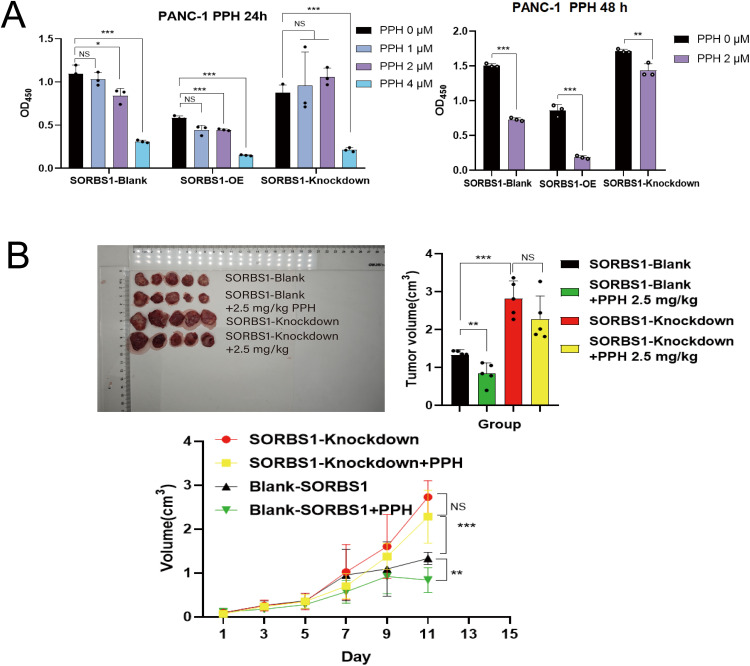
SORBS1 knockdown resisted PPH-induced inhibition in pancreatic cancer *in vivo* and *in vitro*. (A) CCK-8 assay showed the differences in the effects of PPH on SORBS1 with different expression levels at different time points (*n* = 3; 24 h; *p < 0.05, **p < 0.01, ***p < 0.001 compared with PPH 0 μM group; ns, no significance); (B) The difference in the drug effect of PPH in the two groups *in vivo* (*n* = 5; **p < 0.01,***p < 0.001 compared with SORBS1-blank group; ns, no significance)

Given that PPH downregulated SORBS1 expression (Fig. 2B) and that SORBS1 knockdown promoted cell proliferation in pancreatic cancer (Fig. 4B), this study hypothesized that PPH-induced SORBS1 downregulation by PPH could attenuate the restraining influence of PPH on the expansion of pancreatic cancer cells. Thus, this hypothesis was explored *in vitro* and *vivo*. Based on the IC_50_ value of 3.11 ± 0.32 μM PPH for PANC-1 cells (Fig. 1B), the doses were categorized as 2 μM for low and 4 μM for high. The results of the CCK-8 assay indicated that while SORBS1 knockdown reversed the hindering consequence of low-dose PPH on pancreatic cancer viability *in vitro*, no significant influence was noted with high-dose PPH treatment. Comparing the OD_450nm_ values of the experimental groups to their respective control groups, the CCK-8 assay showed that SORBS1 knockdown alleviated the inhibitory effect of 2 μM PPH on PANC-1 cells, whereas the inhibitory effect of 4 μM PPH was not alleviated by the knockdown (Fig. 5A). Considering the superior efficacy of PPH against PANC-1 cells in the experiments depicted in Fig. 1B, PANC-1 cells were selected for *in vivo* experiments. In a nude mice xenograft model, SORBS1 knockdown promoted pancreatic cancer proliferation *in vivo*. Moreover, while tumor volumes were lower in both the PPH-treated SORBS1-blank and the SORBS1-knockdown groups compared with their respective controls, comparing the SORBS1-knockdown + PPH group with the SORBS1-blank group uncovered that SORBS1 knockdown attenuated the inhibitory effect of PPH on pancreatic cancer. Every other day, mice received an intraperitoneal injection of 2.5 mg/kg PPH. After two weeks, the subcutaneous tumors were excised and imaged. PPH reduced tumor volume in both the SORBS1-blank and the SORBS1-knockdown groups compared to their respective controls; however, SORBS1 knockdown reversed the inhibitory effect of PPH on pancreatic cancer when compared to the SORBS1-blank group (Fig. 5B).

### 3.4 SORBS1 Knockdown Mitigated PPH-Induced DNA Damage and Inhibited Apoptosis in Pancreatic Cancer

**Figure 6:**
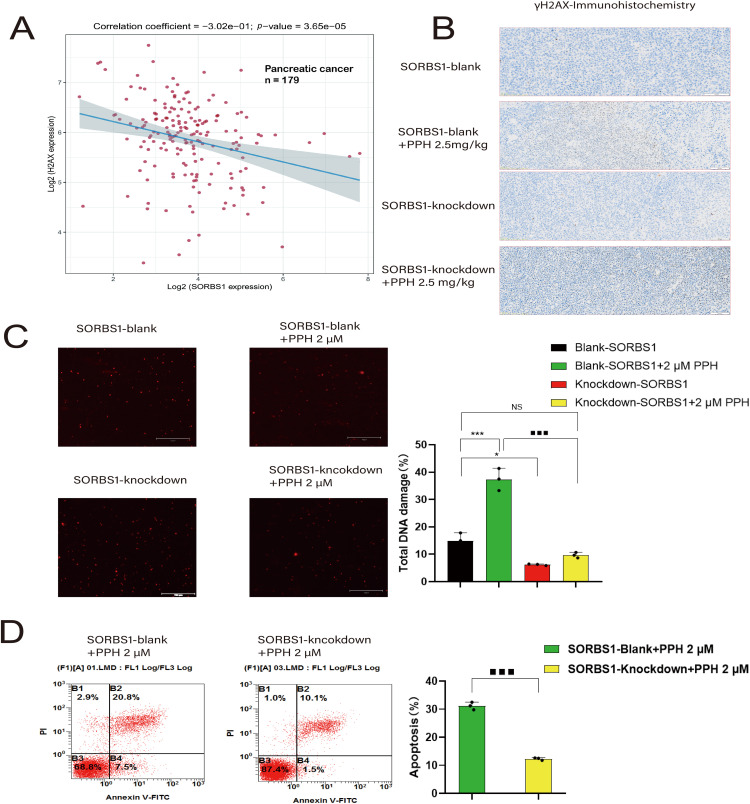
SORBS1 knockdown resisted PPH-induced DNA damage and apoptosis in pancreatic cancer. (A) Scatter plot and fitting line of Spearman correlation analysis between SORBS1 and H2AX expression in pancreatic cancer. Gene expression levels were indicated on the *X*-axis and *Y*-axis, with each point symbolizing a single sample (*n* = 179); (B) Immunohistochemical analysis of γH2AX protein expression (indicated by brown staining) in xenograft tumor tissues from nude mice (*n* = 3); (C) PPH induced DNA damage in PANC-1 cells and SORBS1 knockdown resisted this damage (*n* = 3; *p < 0.05, ***p < 0.001 compared with SORBS1-blank group; ^■■■^*p* < 0.001 compared with SORBS1-blank + PPH group; ns, no significance); (D) SORBS1 knockdown attenuated PPH-induced apoptosis in PANC-1 cells (*n* = 3; ^■■■^*p* < 0.001 compared with SORBS1-blank + PPH group)

Previous studies have established that Paris *polyphylla* saponin increases ROS levels in cancer cells [5,14]. In turn, ROS accumulation leads to extensive DNA damage [15], triggering apoptosis if the damage is irreparable [16,17]. H2AX (H2AFX) is rapidly phosphorylated to generate γH2AX, acting as an indicator of DNA damage in eukaryotic cells [18]. Correlation analysis between SORBS1 and H2AX revealed a correlation coefficient of –0.302 in pancreatic cancer (*n* = 179) (Fig. 6A). Moreover, the immunohistochemical assay unveiled that SORBS1 knockdown down-regulated PPH-induced H2AX expression *in vivo* (Fig. 6B). These observations collectively suggested that SORBS1 knockdown has the potential to alleviate PPH-induced DNA damage. At the same time, the results of the comet assay suggested that SORBS1 knockdown protected PANC-1 cells from PPH-induced DNA damage. Compared to the SORBS1-blank group, the degree of DNA damage in PANC-1 cells was significantly higher in the SORBS1-blank + PPH group. In contrast, compared to the SORBS1-blank + PPH group, the degree of PPH-induced DNA damage was lower in the SORBS1 knockdown + PPH group. In contrast, no significant difference was noted between the SORBS1-blank group and the SORBS1 knockdown + PPH groups. Comet assay showed that PPH induced DNA damage in PANC-1 cells, and SORBS1 knockdown resisted this damage (Fig. 6C). Moreover, compared to the SORBS1-blank + PPH group with the SORBS1 knockdown + PPH group, SORBS1 knockdown significantly attenuated PPH-induced apoptosis in PANC-1 cells, as evidenced by the results of flow cytometry (Fig. 6D).

### 3.5 SORBS1 Knockdown Reversed PPH-Induced S/G2 Arrest in Pancreatic Cancer

**Figure 7:**
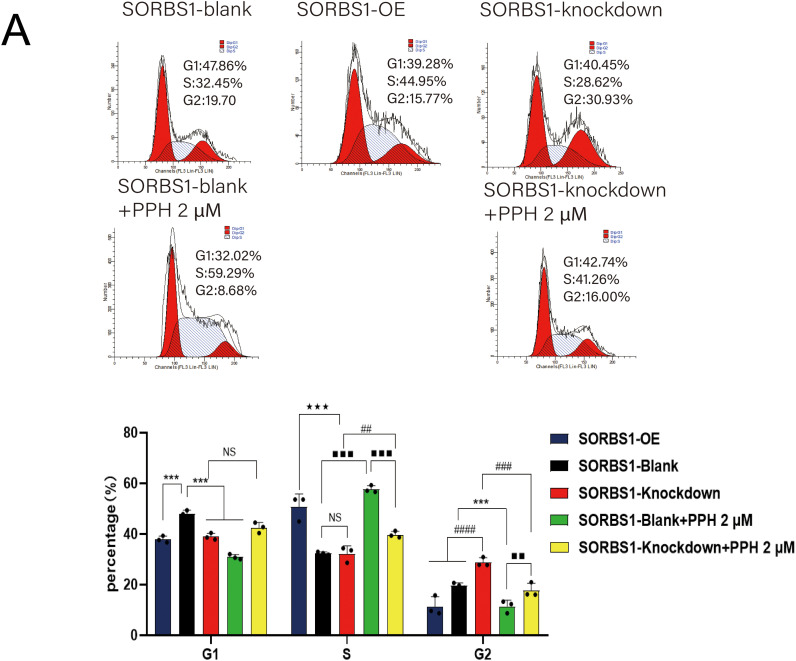

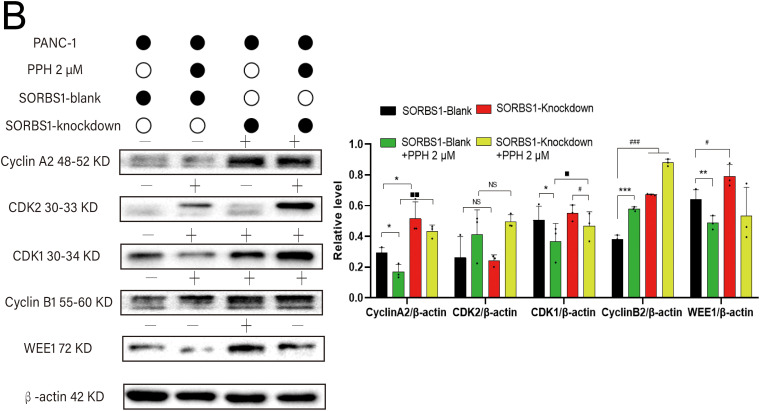
SORBS1 knockdown reversed PPH-induced S/G2 arrest in pancreatic cancer. (A) Flow cytometry analysis revealed that the effects of SORBS1 at different expression levels on cell cycle and the difference in cell cycle arrest between the Blank and Knockdown groups (*n* = 3; ***p < 0.001 compared with SORBS1-blank group; ^★★★^*p* < 0.001 compared with SORBS1-overexpression group; ^##^*p* < 0.01, ^###^*p* < 0.001, ^####^*p* < 0.0001 compared with SORBS1-knockdown group; ^■■^*p* < 0.01, ^■■■^*p* < 0.001 compared with SORBS1-blank + PPH group; ns, no significance); (B) Western blot analysis revealed that the different regulatory effects of PPH on WEE1, Cyclin A2, CDK1 and Cyclin B1 in the Blank group and Knockdown group were observed (*n* = 3; “+” represents positive, and “−” represents negative compared with SORBS1-blank group; “●” represents added and “○” represents not added; *p < 0.05, **p < 0.01, ***p < 0.001, ^#^*p* < 0.05, ^###^*p* < 0.001, ^■^*p* < 0.05, ^■■^*p* < 0.01; ns, no significance)

DNA damage typically activates cell cycle checkpoints, arresting the cell cycle to permit repair or initiating apoptosis if the damage is permanent [16,17]. Given that SORBS1 knockdown resisted PPH-induced DNA damage and inhibited apoptosis in pancreatic cancer, the effect of SORBS1 on the cell cycle in pancreatic cancer was examined. Flow cytometry analysis revealed that PPH induced S/G2 arrest in PANC-1 cells. Compared with the SORBS1-blank group, PPH significantly boosted the proportion of cells arrested in the S/G2 phase. Likewise, compared with the SORBS1 knockdown group, the SORBS1 knockdown + PPH group significantly arrested cells in the S/G2 phase. However, SORBS1 knockdown promoted G2 phase progression, even in the presence of PPH. Compared with the SORBS1-blank group, SORBS1 knockdown significantly promoted S/G2 progression, whereas PPH and SORBS1 overexpression significantly induced S/G2 arrest. Compared with the SORBS1-blank + PPH group, G2 phase progression was significantly promoted in the SORBS1 knockdown + PPH group (Fig. 7A). As an essential regulator, WEE1 manages the G2/M checkpoint in the cell cycle and assists in DNA damage repair before mitosis begins [19]. Moreover, by controlling CDK1 activity, it lengthens the G2 phase, which allows more time for DNA repair [20]. CDK1 regulates the G2-M transition and promotes mitotic entry by binding to cyclin B1 [21]. Cyclin A2, an essential controller of the S/G2 phase transition, associates with CDK2 to promote S phase progression and interacts with CDK1 to drive the G2 phase [22–24]. Western blot analysis revealed that SORBS1 knockdown increased the expression levels of Cyclin A2, CDK1, and WEE1, as well as reversed PPH-induced down-regulation induced by PPH. Compared with the SORBS1-blank group, the expression levels of Cyclin A2, CDK1, and WEE1 were significantly higher in the SORBS1 knockdown and significantly lower in the SORBS1-blank + PPH group. Compared with the SORBS1-blank + PPH group, the expression levels of Cyclin A2, CDK1, and WEE1 were significantly higher in the SORBS1 knockdown + PPH group. Moreover, compared with the SORBS1-blank group, Cyclin B1 expression levels were higher in both the SORBS1 knockdown and SORBS1 knockdown + PPH groups. However, SORBS1 knockdown did not influence CDK2 expression (Fig. 7B). Finally, immunohistochemical staining also displayed that SORBS1 knockdown increased WEE1 expression (Fig. 7C).

## 4 Discussion

Primary and acquired resistance to anticancer drugs remains a significant challenge due to the intricate interactions between drugs and tumors [25]. According to what is currently known, this is the pioneering study to explore the role of SORBS1 in pancreatic cancer. As described earlier, DNA damage generally activates cell cycle checkpoints, stopping the cell cycle to enable repair or starting apoptosis if the damage cannot be fixed [16,17]. According to prior investigations, high-intensity, pan-nuclear γH2AX staining is an indicator of DNA damage [26–28]. More importantly, for DNA damage to be repaired effectively, both WEE1 and an operational checkpoint response are required [29]. As a key regulator, WEE1 is involved in the G2/M cell cycle checkpoint and DNA damage response pathways [30]. To promote the S phase, Cyclin A2, a key regulator of the S/G2 transition, forms a partnership with CDK2 and it associates with CDK1 to drive the G2 phase [22–24]. At the G2/M checkpoint, Cyclin B1 serves as a marker for entry into the M phase, with progression into mitosis being a precisely regulated process ultimately driven by the cyclin B1/CDK1 [31]. Taken together, these results indicated that SORBS1 knockdown activates the G2/M checkpoint, thereby facilitating the repair of PPH-induced DNA damage through WEE1 upregulation. This mechanism prevents apoptosis that could result from irreparable DNA damage and promotes the reinitiation of the G2/M transition by increasing the expression levels of Cyclin A2, CDK1, and Cyclin B1. Consequently, SORBS1 knockdown confers resistance to the inhibitory effects of PPH on pancreatic cancer *in vivo* and *vitro*.

The G2 checkpoint is crucial for compensating for the tumor cells missing p53 show G1 checkpoint deficiency. WEE1 mediates the G2 checkpoint, thus enabling DNA damage fix up. Previous studies have evinced that WEE1 restraint is most valid in p53-mutated cells, wherein the loss of the G1/S checkpoint increases reliance on the G2/M checkpoint [32,33]. Given the high prevalence of p53 mutations in pancreatic cancer, the p53-controlled DNA damage repair checkpoint becomes ineffective, making the WEE1-controlled G2/M checkpoint critically important for pancreatic cancer cell survival. Literature reports indicate that PANC-1 cells harbor p53 mutations [34]. The observations of this study suggest that low SORBS1 expression in PANC-1 cells increases WEE1 activity, contributing to DNA damage repair and facilitating pancreatic cancer cell cycle reinitiation, thus conferring resistance to PPH. These results suggest that the SORBS1-WEE1 axis may are crucial in determining survival and therapy resistance in pancreatic cancer.

The identification of low SORBS1 as a mediator of resistance to PPH highlights the need for elucidating the complex interactions between natural products and cancer. Low SORBS1 levels play a key role in PPH resistance. Understanding these interactions is crucial for developing effective treatments. These findings expand our understanding of the molecular contexts in which natural products can be effectively applied for pioneering more effective therapeutic strategies. However, it is worthwhile acknowledging that this study neither investigated the impact of SORBS1 knockdown on resistance to clinically first-line anticancer drugs nor explored the mechanism by which PPH downregulates SORBS1 expression.

## Data Availability

The data that support the findings of this study are available from the Corresponding Author, Haifeng Tang, upon reasonable request.
